# Simultaneous treatment of a pubovesical fistula and lymph node metastasis secondary to multimodal treatment for prostate cancer: Case report and review of the literature

**DOI:** 10.1515/med-2022-0577

**Published:** 2022-11-01

**Authors:** Giovanni Cochetti, Alessio Paladini, Graziano Felici, Angelica Tancredi, Valerio Cellini, Michele Del Zingaro, Ettore Mearini

**Affiliations:** Department of Medicine and Surgery, Urology Clinic, University of Perugia, Perugia, PG, Italy; Department of Medicine and Surgery, Urology Clinic, University of Perugia, Piazzale Giorgio Menghini, 1, 06129, Perugia, PG, Italy

**Keywords:** pubovesical fistula, prostate cancer, radical cystectomy, lymphadenectomy

## Abstract

Pubovesical fistula (PVF) is a rare complication of radical treatments for prostate cancer (PCa), especially when a multimodal approach is performed. We present a case of PVF with extensive communication between the bladder and the pubic bones, and lymph node metastases of PCa treated by cystectomy and salvage lymphadenectomy. We describe a case of a 65-year old male patient who, after radical prostatectomy and adjuvant radiation therapy, suffered from suprapubic and perineal pain, ambulation difficulties and recurrent urinary tract infections. Cystoscopy, cystography and contrast-enhanced magnetic resonance imaging diagnosed a PVF. Choline positron emission tomography/computed tomography scan demonstrated PCa lymph node metastases. After the failure of conservative treatment, open radical cystectomy with ureterocutaneostomy diversion and salvage lymphadenectomy were performed with resolution of symptoms. At 3-month follow-up, the pelvic and perineal pain was completely regressed and 1-year later the patient was still asymptomatic. This clinical case shows efficacy and safety of combined salvage lymphadenectomy and cystectomy with urinary diversion for the treatment of late PCa node metastasis and PVF.

## 1 Introduction

Prostate cancer (PCa) is the second most common cancer and the fifth leading cause of cancer death in men worldwide. It is estimated that around 1.4 million cases of PCa were diagnosed and there were 375,000 associated deaths worldwide in 2020 [1,2]. Radical prostatectomy (RP) and radiation therapy (RT) are the standard treatments for organ-confined low-risk and intermediate PCa [3]. In case of high-risk and locally advanced PCa a multimodal treatment is required. If the most frequent side effects of PCa treatments are urinary incontinence and erectile dysfunction [4,5,6,7], however, especially when a multimodal treatment is needed, some rare complications may arise including the pubovesical fistula (PVF). This one is an infrequent but distressing complication affecting men’s quality of life (QoL). PVF could be characterized by recurrent urinary tract infections, symphysis pubis pain due to osteitis or osteomyelitis, intractable pelvic pain and walking difficulties. Because of the rarity of PVF, no well-established algorithm or guideline exists for its treatment [8,9,10,11].

The aim of this study was to describe the management of a PCa lymph node metastasis and a rare case of PVF secondarily to RP, adjuvant RT and, subsequently, incision of vesicourethral anastomosis stenosis and trans-urethral resection of a bladder tumor (TURBT). A narrative review of the literature concerning the management of PVF was also carried out. We present the following case in accordance with the CARE reporting checklist.

## 2 Case presentation

A 65-year old Caucasian male underwent robotic non-nerve sparing RP and bilateral extended lymphadenectomy for a high-risk PCa. Definitive histological examination showed a prostate adenocarcinoma ISUP 5 (Gleason score 4 + 5) with positive surgical margin (pT3a N0 R1). Adjuvant RT with 76 Gray and 3 years of androgen deprivation therapy (ADT) were administered to complete the multimodal treatment. Two years after the accomplishment of the multimodal therapy a biochemical recurrence due to a lymph node metastasis occurred. Hormone therapy with Leuprorelin and subsequent addition of Enzalutamide were administered obtaining a decrease of prostate-specific antigen (PSA) level from 6.4 to 1.58 ng/mL.

After 2 years, the patient underwent TURBT in his referred center for a bladder cancer located in the anterior wall and bladder dome; the histological examination showed a non-invasive low-grade urothelial cancer. After 2 years, an endoscopic incision of vesicourethral anastomosis stenosis and ablation of a Hem-o-lok there migrated were performed. Few months after surgery, the patient suffered from suprapubic and perineal pain non-responding to analgesic therapy, ambulation difficulties and recurrent urinary tract infections sustained by *Escherichia coli*. With the clinical suspect of a PVF, an indwelling catheter was placed without success for continuous inflate balloon rupture and pelvic pain.

Afterward, the patient was addressed to our center. QoL and painful symptoms were evaluated using the Quality-of-Life Scale (QOLS) and the Visual Analog Scale (VAS) with a value of 25 and 9, respectively [12].

**Figure 1:**
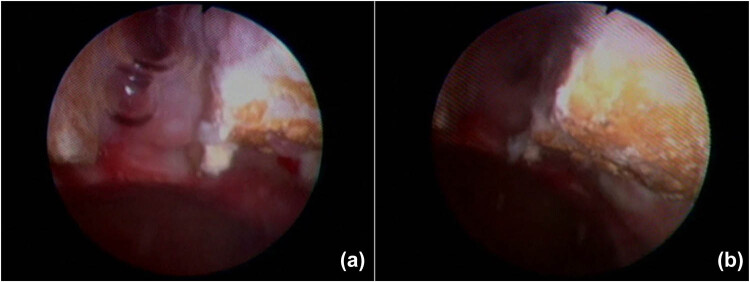
Cystoscopy shows a suspected PVF at the 12 o’clock position of the bladder neck, close to the center of the eroded pubic symphysis (a and b).

**Figure 2:**
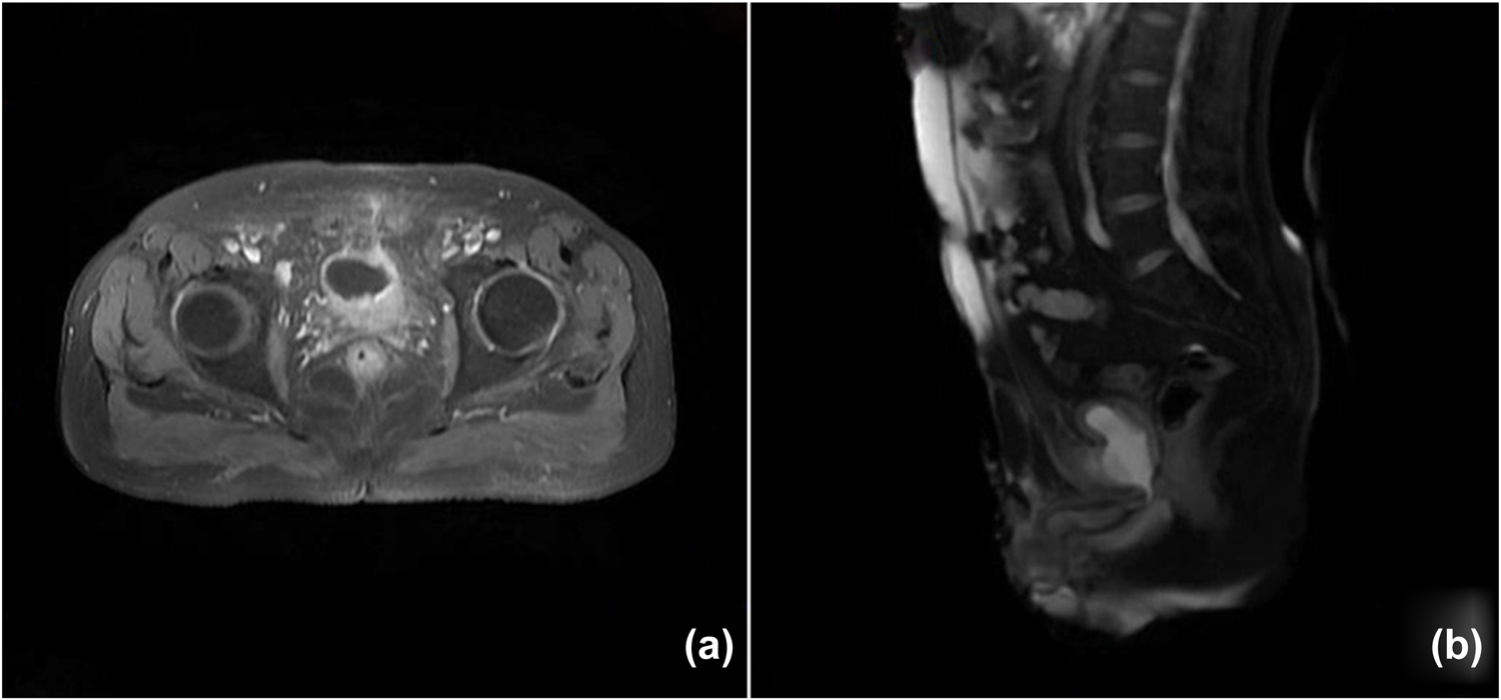
MRI of the pelvis confirming the presence of pubovesical fistula. Axial (a) and sagittal (b) images.

After a cystoscopy and cystography showing a suspected PVF at the 12 o’clock position of the bladder neck, close to the center of the eroded pubic symphysis, without any evidence of contrast medium spillage (Figure 1), a suprapubic catheter was placed. An abdominal magnetic resonance imaging (MRI) confirmed the presence of the fistula with marked edema of the surrounding soft tissues, which mainly involved the medial sectors of the external obturator muscles and the anterior portion of the internal obturators; considerable edema also involved the spongy bone of both pubic branches (Figure 2).

**Figure 3:**
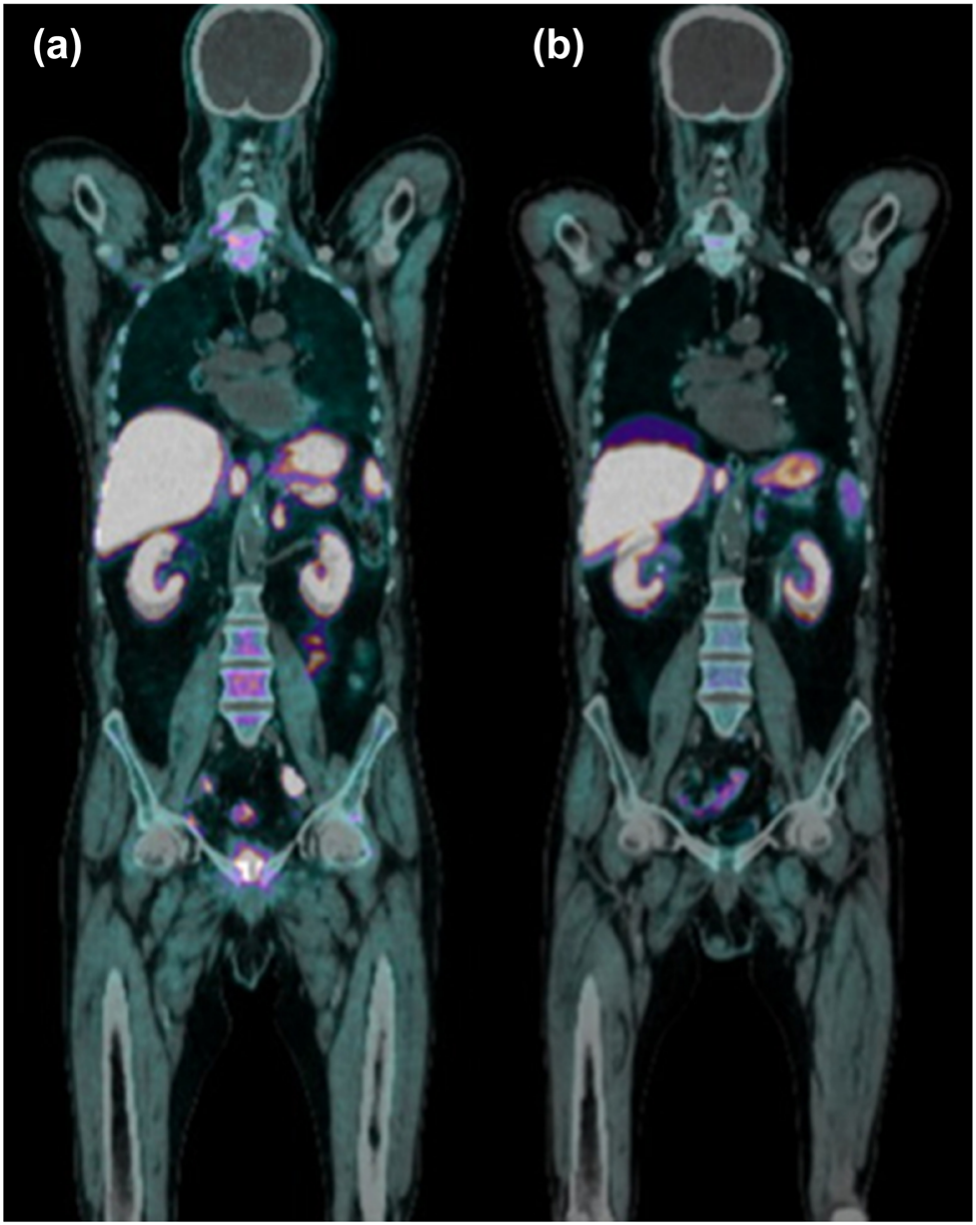
Choline PET-CT detects a left external iliac lymphadenopathy and a further capture of the tracer at the level of other left external iliac lymph nodes and inguinal lymph nodes bilaterally (a). Choline PET-CT performed after cystectomy and lymphadenectomy where previously highlighted lymphadenopathies are no longer visible (b).

Furthermore, due to biochemical recurrence (PSA level 0.78 ng/mL), a choline positron emission tomography/computed tomography (PET/CT) scan was carried out, detecting a 20 mm left external iliac lymphadenopathy and a further capture of the tracer at the level of other left external iliac lymph nodes, and inguinal lymph nodes bilaterally. In addition, PET/CT showed a “ribbon-like activity” stretching from the anterior region of the bladder to the pubic symphysis consistent with the fistulous conduit (Figure 3).

Despite the epicystostomy, the PVF and related painful syndrome persisted. For this reason, the patient underwent open radical cystectomy with extended lymphadenectomy and bilateral ureterocutaneostomy due to fibrosis of the terminal tract of both ureters at the frozen section. During surgery, the diastasis of pubic symphysis was evident and the bone tissue had eroded the bladder wall at the bladder neo-neck level. The operative time was 200 min with 400 mL of estimated blood loss. The length of hospital stay was 10 days. Definitive histological findings showed chronic follicular cystitis of the bladder and extended and poorly differentiated adenocarcinoma metastases consisting with PCa at the level of the left external iliac lymph node.

At one postoperative month, the painful syndrome was significantly improved with QOLS and VAS of 50 and 5, respectively. At 3-month and 1-year follow-up, the pelvic and perineal pain was completely regressed and the patient was satisfied with the outcome of the surgical treatment and his QoL.

Serum PSA was 0.01 ng/mL without hormonal therapy at first postoperative month and it remains not evaluable at 1-year follow-up and the post-operative choline PET/TC was negative for lymph node metastases.

The study was conducted in accordance with ethical principles of the Helsinki declaration. The informed consent was obtained from the patient. Written informed consent was obtained from the patient for publication of this case report and any accompanying images.


**Ethical statement:** All procedures performed in studies involving human participants were in accordance with the ethical standards of the institutional and/or national research committee(s) and with the Helsinki Declaration (as revised in 2013). Written informed consent was obtained from the patient for publication of this case report and accompanying images. A copy of the written consent is available for review by the editorial office of this journal.

## 3 Discussion

In recent years, the incidence of early-onset PCa has increased and is often associated with a high degree of malignancy and advanced stage, leading to a worst prognosis than late-onset PCa. In case of high-risk and locally advanced PCa, a multimodal treatment increases the complication rate.

PVF is a rare complication of the PCa treatment such as RP, external-beam radiation therapy (EBRT), cryotherapy or high-intensity focused ultrasound (HIFU), especially when multiple treatments are required (Table 1). Prior endoscopic procedures, such as incision or dilatation of bladder neck, are a risk factor for urinary tract infections and PVF, resulting osteomyelitis.

**Table 1:** Pubovesical fistula after PCa treatment – literature review

Authors (year)	Number of cases	Type and % PCa treatment	Conservative treatment of PVF (%)	Surgical reconstruction (%)	Cystectomy and urinary diversion (%)	Resolution of symptoms (%)	Complications (%)	Recurrence (%)
Matsushita et al. (2012) [9]	12	3 EBRT (25%)	1 (8.3%)	None (0%)	11 (91.6%)	12 (100%)	/	/
		5 EBRT and following salvage RP (41.7%) 4 RP and salvage RT (33.3%)						
Moore et al. (2013) [30]	1	Robotic RP + salvage pelvic irradiation	None	None	Cystectomy with orthotopic neobladder and anterior pelvic resection	100%	None	None
Bugeja et al. (2015) [10)	16	8 (50%) RP and salvage RT	1 (6.25%)	7 (43.75%)	8 (50%)	15 (93.75%)	9 (56.25%)	1 (6.25%)
		5 (31.3%) various combinations of brachytherapy, EBRT and cryotherapy					1 death from respiratory complication	
		3 (18.7%) salvage cryotherapy after radiotherapy (EBRT or brachytherapy)					1 prolonged leak from anterior bladder wall	
							1 osteomyelitis	
							3 prolonged ileus	
							1 wound infection	
							1 chest infection	
							1 chest infection and pelvic collection	
Lavien et al. (2017) [8]	20	/	3 (18.75%)	None (0%)	17 (85%)	19 (95%)	/	/
			2 antibiotic therapy					
			1 suprapubic catheter					
Osterberg et al. (2017) [31]	31	23 (74.1%) primary radiotherapy:	None (0%)	12 (38.7%)	19 (61.2%)	/	18 (58%) postoperative ileus	1 (3.2%)
		13 EBRT					13 (41.9%) postoperative infections	
		5 brachytherapy						
		5 brachytherapy + EBRT						
		5 (16.1%) RP + RT						
		2 (6.5%) RP + salvage RT						
		1 proton beam radiotherapy (3.2%)						
		18 (58%) endoscopic procedure						
Shapiro et al. (2018) [32]	5	5 (100%) EBRT	None (0%)	None (0%)	5 (100%) and contextual pubic debridement	3 (60%)	1 (20%) pubic osteomyelitis and death	1 (20%)
		5 (100%) endoscopic procedure of incision or dilatation of bladder neck						
Navaratnam et al. (2019) [13]	12	4 (33.3%) RP and salvage EBRT	None (0%)	None (0%)	11 (91.7%)	11 (91.7%)	1 (8.3%) Pelvic abscess	2 (16.7%) Recurrent osteomyelitis
					1 (8.3%)			
		3 (25%) EBRT and HDR Brachytherapy			Only robot-assisted Ho:YAG laser debridement			
		2 (16.6%)						
		EBRT and salvage cryotherapy						
		1 (8.3%) EBRT and salvage HIFU						
		1 (8.3%) LDR brachytherapy						
		1 (8.3%)						
		Urosymphyseal fistula after PVP						
Nosé et al. (2020) [33]		21 (58.3%) Prior prostatectomy	0%	1 (2.8%)	35	36 (100%)	None (0%)	None (0%)
	36				(97.2%)			
		31 (86.1%)						
		Prior EBRT						
		11 (30.6%)						
		Prior brachytherapy						
		9 (25.0%)						
		Brachytherapy + EBRT						
		29 (80.6%)						
		Prior endoscopic procedure						
Kahokehr et al. (2021) [34]	36	33 (91.7%) prior radiation:		1 (2.8%) no prior RT	33 (91.7%)	34 (94.4%)	/	/
		21 (58.3%) EBRT						
		10 (27%) EBRT + brachytherapy						
		2 (5.5%) brachytherapy						
		21 (58.3%) Prior prostatectomy						
		29 (80.6%)						
		Prior endoscopic procedure						
		1 (2.8%) salvage cryotherapy						
		1 (2.8%) HIFU						
		2 (5.6%) prior cystectomy and urinary diversion						
Andrews et al. (2021) [27]	25	5 (20%) RP + adjuvant EBRT	None (0%)	1 (4%)	22 (88%)	23 (92%)	12 (48%)	/
					While 2			
		10 (40%) RP + salvage EBRT			8%			
		3 (12%) EBRT + salvage cryotherapy			Salvage RP			
		1 (4%) EBRT + salvage brachytherapy						
		3 (12%) EBRT						
		2 (8%) brachytherapy						
		1 (4%) brachytherapy + EBRT						

PVF consists of a direct communication between the urinary tract and pubic symphysis. A similar condition, a puboprostatic fistula, can also occur after treatment for benign prostatic hyperplasia such as transurethral resection of the prostate or photoselective vaporization of the prostate (PVP) [9,13,14,15,16]. Clinical symptoms and signs of PVF are pubic pain and discomfort, ambulation difficulties, recurrent urinary tract infections, pubic symphysis osteitis and osteomyelitis. Generally, these symptoms negatively affect the patient’s QoL [6,9]. Diagnosis is possible by imaging examinations such as cystography, CT or MRI with medium contrast. In particular, MRI seems to be more sensitive and more specific than CT in detecting PVF, especially when associated with osteomyelitis [6,17,18]. However, cystoscopy can be a useful examination to evaluate the presence and location of the fistula and to plan the therapeutic strategy.

The first-line therapy is a conservative management with antibiotic therapy, anti-inflammatory drugs and urinary diversion through bladder catheter, epicystostomy or nephrostomies. However, the failure of conservative treatment is reported in 75–94% of these patients and the surgical intervention is almost always necessary [19].

In most cases, surgery consists of radical cystectomy with urinary diversion and possible debridement for osteomyelitis if feasible and required. In fact, unlike rectovesical fistulas, for the most of PVFs there is no possibility of a conservative approach. After cystectomy, almost all patients report the disappearance or sharp reduction of pelvic pain. Recently, Matsushita et al. reported that 10 out 12 patients affected by PVF, arised after pelvic radiotherapy and bladder neck incision, resolved the symptoms after radical cystectomy [9].

Although open radical cystectomy with urinary diversion and pubic symphyseal debridement is the standard surgical strategy, recently the safety and efficacy of the robotic approach have also been demonstrated. Navaratnam et al. demonstrated the efficacy and safety of holmium laser debridement of the pubic bone with concomitant robot-assisted cystectomy (RARC) and urinary diversion in patients with PVF and pubic symphysis osteomyelitis. The authors found, at the median follow-up of 29 months, 11 out of 12 patients with PVF underwent RARC had complete resolution of pelvic symptoms [13].

Recently, Bugeja et al. described their experience in treating 16 patients with PVF. Among these, eight had undergone RT as primary treatment and the other eight had undergone RP. In this study, conservative therapy was resolutive only for one patient, eight patients underwent cystectomy with ileal conduit diversion using an open approach and seven patients underwent salvage RP and surgical reconstruction with excision of the fistulous tract and involved symphyseal bone, drainage of cavities and omentoplasty. All the patients reported the resolution of symptoms [10].

In our case, the fistula occurred after multimodal therapy for PCa: RP and adjuvant RT on prostatic lodge, TURB and endoscopic incision of anastomosis. Unlike the other reported PVFs, the communication between bladder and symphysis was so extensive to be described as a penetration of pubic symphysis into the bladder. For this reason, after the failure of the conservative therapy, a radical cystectomy with ureterocutaneostomy was performed using an open approach. After the surgery, the patient immediately reported a significant improvement of painful symptoms until their disappearance after 3 months.

In our study, we evaluated the symptoms and, especially, the pelvic pain by validated scores. The improvement of the symptoms was highlighted by the QOLS, which increased from 25 before the surgery to 85 at 3 months after the cystectomy as well as the VAS, which decreased from 9 to 1.

Another relevant aspect in this clinical case is that the radical cystectomy was combined with salvage bilateral lymphadenectomy for lymph node recurrence of PCa. In particular, a total of 15 lymph nodes were removed and only one, located in the left external iliac resulted in a metastasis of poorly differentiated PCa. In this area, choline PET/CT had showed a pathologic hyperfixation of about 20 mm. After lymphadenectomy with pathological lymph node asportation and interruption of hormone therapy with Leuprorelina and Enzalutamide, the PSA value after 1 month decreased from 1.58 to 0.01 ng/mL.

The treatment of recurrent PCa after RP is conventionally carried out with RT and ADT. However, in recent years the concept of treating oligometastatic disease and in particular nodal recurrent PCa after radical treatments with salvage lymphadenectomy is a topic of debate [20,21]. This is possible thanks to new diagnostic methods, such as choline PET/CT, and even better with PSMA PET/CT, which allows an early identification of lymph node recurrent disease [22,23]. Several studies assessed the outcomes of salvage lymphadenectomy in patients with lymph node metastases after RP. Suardi et al. reported the experience of 59 patients treated with lymphadenectomy for nodal recurrent PCa [24]. Among these, 59.3% had a biochemical response and the 8-year biochemical recurrence-free survival rate in patients with complete biochemical response was 23%. However, at 8 years, clinical recurrence-free survival and cancer-specific mortality free survival were 38 and 81%, respectively. The authors also identified PSA at surgery, time to BCR and involvement of retroperitoneal nodes as risk factors for biochemical recurrence [20,24]. Herlemann et al. reported that 29.8% of the patients developed complete biochemical response and 56.7% partial biochemical response after salvage metastatic lymph node dissection. The 5-year biochemical recurrence free was 6.2%, clinical recurrence-free was 26.0% and cancer-specific survival rate was 82.8% [25].

Currently, the salvage extended pelvic lymphadenectomy could be justified for patients with “node-only” PCa recurrence, with PSA level <4 ng/mL and Gleason score ≤7. This procedure may be useful to postpone hormonal treatment. However, studies and longer follow-up studies are necessary to verify the effectiveness of this approach [26,27,28,29].

The main limit of this study is related to its nature of being a case report. For this reason, we cannot state a conclusive recommendation about the best treatment of PVF.

In conclusion, we described a case the PVF due to multimodal therapy for PCa. This clinical case is particularly interesting because of the extensive communication between the bladder and the pubic bones, which can be defined as a penetration of the pubic symphysis into the bladder, not yet reported in the literature. Interestingly, we proposed radical cystectomy with urinary diversion combined with salvage extensive lymphadenectomy as treatments for PVF and recurrent PCa, respectively.
